# Nitroxoline revisited: antibacterial mechanisms, spectrum, and therapeutic potential of an old drug in the era of antimicrobial resistance

**DOI:** 10.1042/BST20250045

**Published:** 2026-07-09

**Authors:** Elisabetta Cacace, Stephan Göttig

**Affiliations:** 1 Department of Biology, Institute of Microbiology and Swiss Institute of Bioinformatics, ETH Zurich, Zurich, Switzerland; 2 Institute for Medical Microbiology and Infection Control, University Hospital, Goethe University Frankfurt, Frankfurt am Main, Germany; 3 University Center for Infectious Diseases (UCI), University Hospital, Goethe University Frankfurt, Frankfurt am Main, Germany

**Keywords:** antibiotic resistance, antibiotics, bacterial infections, drug repurposing

## Abstract

As the spread of antimicrobial resistance outpaces antibiotic discovery and marketing, there is a critical need to revisit approved compounds with untapped potential. Metal-based complexes (metallo-antimicrobials) represent ideal candidates for such efforts due to their pleiotropic effects, which, unlike antibiotics with single protein targets, may have lower resistance potential. With its well-characterised safety profile, nitroxoline, an 8-hydroxyquinoline derivative, is a prominent compound of this kind. Here, we review recent advances in our understanding of nitroxoline’s multimodal mode of action, focusing on its disruption of metal homeostasis at the intracellular and extracellular level. We further explore its clinical utility, including its low propensity for resistance development, and its potential for both combinatorial therapy and as an anti-biofilm agent. Although beyond the scope of the present review, we refer to nitroxoline’s antifungal effects whenever they represent a relevant comparison with its antibacterial activity. We highlight current knowledge gaps in nitroxoline’s mechanism and *in vivo* activity that should be bridged to accelerate its repurposing, particularly in the treatment of multidrug-resistant and biofilm-associated infections. Several recent reviews have examined nitroxoline from chemical, clinical, and repurposing perspectives [1, 2, 3, 4]. The present work complements these by providing a mechanistic synthesis of nitroxoline’s effects in bacteria, linking its multimodal mode of action to the knowledge gaps that should be addressed before clinical repurposing can proceed.

## Chemistry, mechanism, and spectrum of action

**Figure 1:**
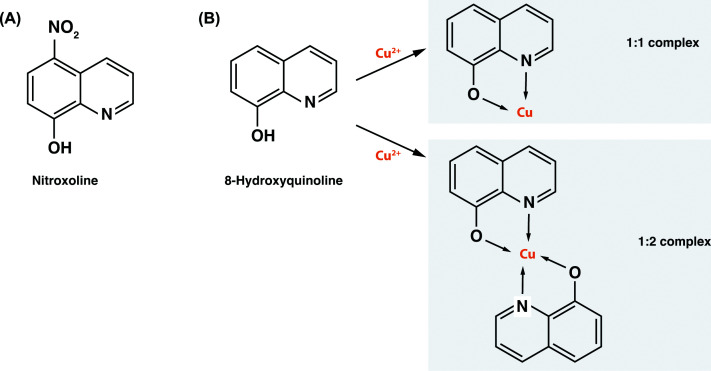
Nitroxoline and 8-hydroxyquinoline structure and metal complex (**A**) Nitroxoline structure. (**B**) 8-Hydroxyquinoline scaffold and its 1:1 or 1:2 metal-to-ligand complex (copper is shown as an example).

Nitroxoline (8-hydroxy-5-nitroquinoline) is a small, lipophilic molecule belonging to the 8-hydroxyquinoline family (Figure 1A). The 8-hydroxyquinoline scaffold is a well-established bidentate chelator of divalent metal cations, coordinating metals through the phenolic oxygen and ring nitrogen [5,6] (Figure 1B). The nitro group also enhances lipophilicity, allowing the molecule to bypass the outer membrane (OM) of Gram-negative pathogens through passive diffusion [7].

Although nitroxoline entered clinical use in the 1960s, the first mechanistic studies appeared only between the 1980s and 1990s. These studies showed that uptake in *Escherichia coli* is magnesium-dependent but energy-independent, with biphasic kinetics, consisting of a rapid initial association followed by a slower increase to a plateau [8]. Mg^2+^ and Mn^2+^ (but not Ca^2+^) alleviated its antibacterial activity, with complex stability following the order Mn^2+^ > Mg^2+^ > Ca^2+^ [8]. These effects were strongly pH-dependent, with increased activity under acidic conditions, indicating that the protonation state modulates chelation efficiency and membrane interactions [8]. In particular, the electron-withdrawing nitro group (-NO_2_) at C-5 lowers the pKa of the C-8 hydroxyl group to ∼6.0, ensuring high chelating activity in acidic environments such as urine [8].

**Figure 2:**
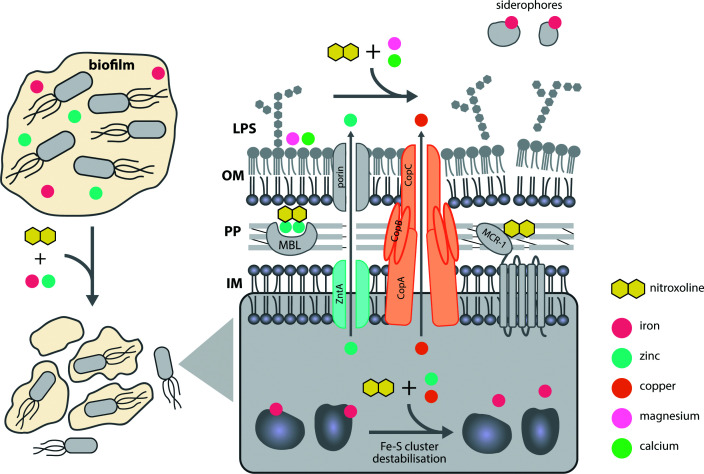
Multifaceted antibacterial mechanisms of nitroxoline against bacteria Nitroxoline exerts antibacterial activity through multiple, interconnected mechanisms centred on its ability to form complexes with metals. Nitroxoline chelates iron and zinc from the biofilm extracellular matrix, reducing biofilm mass and converting compact biofilm architecture to reticulate, loosely organised structures [30]. Nitroxoline chelates divalent cations (Mg^2+^, Ca^2+^) that stabilise LPS cross-bridges in the OM, compromising membrane integrity and increasing permeability [12,14]. Nitroxoline sequesters zinc from MBLs [14,47], hindering their function, and directly interacts with MCR-1 [43], affecting beta-lactam and polymyxin resistance, respectively. Nitroxoline can shuttle copper and zinc into bacterial cytoplasm and induce toxic accumulation of these metals [12]. The resulting intracellular metal stress disrupts Fe-S clusters and metal homeostasis. This induces defensive responses, such as copper and zinc efflux, including the CopABC complex, ZntA and porins, and an increase of siderophores [12]. IM, inner membrane; LPS, lipopolysaccharide; MBL, metallo beta-lactamase; MCR-1, mobilised colistin resistance 1; OM, outer membrane; PP, periplasm.

Nitroxoline’s chelation of divalent cations, including Mg^2+^ and Ca^2+^, likely contributes to its disruption of the OM. This was first shown indirectly by increased surface hydrophobicity, inhibition of bacterial mannose-sensitive hemagglutination [9], and decreased adherence to epithelial cells and catheters [10,11]. This action has been more recently demonstrated in *E. coli* [12] and *Pseudomonas aeruginosa* [13] and leveraged to develop ASN-1733, a long-lasting nitroxoline derivative that competes for Ca^2+^ on the bacterial surface, causing detachment of the lipopolysaccharide (LPS) and compromising OM integrity [14] (Figure 2).

Recent evidence in *E. coli* has highlighted more pervasive effects of nitroxoline on bacterial metal homeostasis beyond the chelation of divalent metal cations, increasing copper and zinc intracellular concentrations [12] (Figure 2). The role of copper in nitroxoline’s antibacterial activity has been confirmed in methicillin-resistant *Staphylococcus aureus* (MRSA), *in vitro* and in *in vivo* infection models, where copper enhanced nitroxoline’s antibacterial activity by promoting aerobic glycolysis [15]. Like other 8-hydroxyquinoline derivatives [16], nitroxoline has also been proposed as a copper ionophore in *Mycobacterium tuberculosis*. Notably, the drug is only effective within infected macrophages where enough copper is available [17].

The activity of nitroxoline as a copper and zinc ionophore would also mirror that of clioquinol, an antimicrobial 8-hydroxyquinoline that reduces disease-associated behaviours in Alzheimer’s animal models by alleviating copper and zinc deficiencies [18]. However, the interaction with copper is not consistently demonstrated across all species and all derivatives. In fungi, nitroxoline decreases copper importers, consistent with copper intoxication as observed in *E. coli* [12], yet copper supplementation antagonises its activity [19]. Future studies should clarify the dependency of such copper-quinoline interplay on the organism, quinoline analogue and copper form considered.

The downstream consequences of these perturbations, beyond the perturbation of OM integrity, are not fully characterised: a consequence of copper and zinc overload could be the damage of proteins containing Fe-S clusters, suggested by their destabilisation by nitroxoline [12]. This is documented in several bacterial species as direct iron displacement by copper or zincb in the cluster [20,21] or targeting of Fe-S cluster assembly proteins [22].

The damage to Fe-S clusters could also explain typical iron deprivation responses observed in *E. coli* upon nitroxoline treatment, despite normal iron levels, as demonstrated by nano-X-ray fluorescence [12]. These effects include upregulation of proteins responsible for Fe-S cluster biosynthesis (with corresponding deletion mutants more sensitive to nitroxoline), as well as elevated intracellular manganese concentrations [12], which can serve as an iron surrogate upon iron scarcity. Therefore, we propose that these changes should be interpreted as responses to copper- and zinc-induced damage of Fe-S clusters, as reported in *M. tuberculosis* for other 8-hydroxyquinoline derivatives [16]. Collectively, these observations support a multimodal mechanism of action, combining metal deprivation and intoxication with downstream pleiotropic effects.

A further proposed mechanism—the inhibition of RNA polymerase—remains incompletely supported in bacteria: evidence is limited to *in vitro* assays using the purified enzyme from *E. coli* [23] and functional assays in yeast, with no direct demonstration in bacteria [24]. Such activity would be a plausible consequence of nitroxoline chelation of Mg^2+^ and Mn^2+^, which are essential cofactors for RNA polymerase function. However, direct functional evidence in bacteria is needed to confirm this mechanism.

Although non-antibacterial effects of nitroxoline fall beyond the scope of the present review, we note that mechanistic studies in amoebae have revealed additional effects—including mitochondrial dysfunction, reactive oxygen species generation, programmed cell death, and DNA damage [25–27]—suggesting that nitroxoline’s mode of action may be broader than the metal-chelation framework characterised in bacteria, and meriting future investigation in prokaryotic systems.

Collectively, these mechanisms underpin a broad antibacterial spectrum, described below.

### Antimicrobial spectrum and bacteriostatic versus bactericidal activity

Nitroxoline exhibits broad *in vitro* activity against Gram-positive and Gram-negative bacteria (Table 1), as well as fungi, including *Aspergillus* and *Candida* species, which fall beyond the scope of the present review.

**Table 1:** Summary of nitroxoline antibacterial activity indicating the bacterial species, type of effect tested, and relevant publications

Species/group	Tested activity (static/cidal)	MIC_50_/_90_ or MIC range (mg/l)	MBC (mg/l)	Key findings or isolate numbers	References
*Achromobacter xylosoxidans*	Bacteriostatic	4/16	–	18 isolates tested	[12]
*Acinetobacter baumannii* (including CRAb)	Bactericidal (concentration-dependent)	2/4	≥1 dilution above MIC	Excellent activity despite carbapenem, ciprofloxacin, and TMP/SMX resistance; biphasic killing with optimal bactericidal concentration	[12,22,33]
*Acinetobacter johnsonii*	Bacteriostatic	2/2	–	13 isolates tested	[12]
*Acinetobacter junii*	Bacteriostatic	2/4	–	8 isolates tested	[12]
*Acinetobacter lwoffii*	Bacteriostatic	2/2	–	6 isolates tested	[12]
*Acinetobacter pittii*	Bacteriostatic	2/4	–	14 isolates tested	[12]
*Acinetobacter ursingii*	Bacteriostatic	1/2	–	8 isolates tested	[12]
*Burkholderia cenocepacia*	Bacteriostatic	16/32	–	14 isolates tested	[12]
*Citrobacter freundii*	Bacteriostatic	8/8	–	41 isolates tested	[12]
*Citrobacter koseri*	Bacteriostatic	4/16	–	18 isolates tested	[12]
*Enterobacter cloacae*	Bacteriostatic	8/16	–	36 isolates tested	[12]
*Enterococcus faecalis*	Bacteriostatic; membrane damage at higher concentrations	MIC 2-16 (strain-dependent)	32 (>64 in 5/29 strains tested)	Synergy with gentamicin (8/29 strains); complete inhibition of adhesion with certain combinations	[46]
*Escherichia coli* (UPEC, including ESBL)	Bacteriostatic (species-dependent bactericidal at higher concentration)	4/8 (Yi et al.); ≤16 (EUCAST BP)	–	Broad activity maintained; low resistance; confirmed bacteriostatic in urine	[8,12,32,33]
*Hafnia alvei*	Bacteriostatic	2/8	–	4 isolates tested	[12]
*Klebsiella oxytoca*	Bacteriostatic	8/8	–	20 isolates tested	[12]
*Klebsiella pneumoniae*	Bacteriostatic	8/32	–	Activity against MDR strains; resistance mutations impose severe fitness costs	[12,33,42]
*Morganella morganii*	Bacteriostatic	16–32	–	22 isolates tested	[12]
*Mycobacterium abscessus* complex	Active *in vitro*	MIC_90_ = 4	–	Activity against drug-resistant isolates	[35]
*Mycobacterium bovis* BCG	Bacteriostatic (10 μM); moderate bactericidal activity at higher concentrations	MIC ∼10 μM (∼2 mg/l)	–	Activity against hypoxic dormant bacilli—relevant for latent tuberculosis	[36]
*Mycobacterium tuberculosis* complex (including MDR)	Bacteriostatic (bactericidal at higher concentration)	MIC_90_ = 4	–	All isolates well below EUCAST *E. coli* BP (≤16); activity maintained against MDR tuberculosis	[34]
*Neisseria gonorrhoeae*	Bacteriostatic	8/8	–	18 isolates tested	[12]
*Proteus mirabilis*	Bacteriostatic	8/16	–	Included in a panel of uropathogens isolated in China	[12,33]
*Proteus vulgaris*	Bacteriostatic	4/8	–	9 isolates tested	[12]
*Pseudomonas aeruginosa*	Intrinsically resistant (planktonic); biofilm susceptible	>16 (most isolates)	–	Not susceptible in planktonic form; sub-MIC biofilm eradication up to 80%; synergy with colistin against colistin-resistant strains	[12,13,33,39]
*Pseudomonas putida*	Bacteriostatic	16/32	–	11 isolates tested	[12]
*Salmonella* spp.	Bacteriostatic	4/4	–	11 isolates tested; also effective against intracellular bacteria	[12]
*Serratia marcescens*	Bacteriostatic	16/32	–	28 isolates tested	[12]
*Staphylococcus aureus* (MRSA)	Biofilm eradication; persister cell killing	MBEC ∼188 μM	–	Active against MRSA persister cells where vancomycin and daptomycin fail; broad-spectrum biofilm eradication	[30]
*Stenotrophomonas maltophilia*	Bacteriostatic	4/16	–	21 isolates tested	[12]

Abbreviations: BP, breakpoint; CRAb, carbapenem-resistant *A. baumannii*; ESBL, extended-spectrum β-lactamase; MBEC, minimum biofilm eradication concentration; MDR, multidrug-resistant; MDR-TB, multidrug-resistant tuberculosis; OM, outer membrane; TMP/SMX, trimethoprim/sulfamethoxazole; UPEC, uropathogenic *E. coli*.

Nitroxoline is active against *Enterobacteriaceae* with minimal inhibitory concentration (MIC) range of 2–16 μg/ml [12,28] and *Moraxellaceae*, including *Acinetobacter baumannii* [29,30], with MICs of 0.5–2 μg/ml [12]. In contrast, activity against *P. aeruginosa* [7] and *Burkholderia cepacia* complex [12] is more limited (MICs ranging from 16–32 and 16 μg/ml, respectively). Gram-positive species are broadly susceptible, including *Staphylococcus aureus* (MIC 8 μg/ml) and *Listeria monocytogenes* (MIC 1 μg/ml) [7]. Notably, nitroxoline retains activity against stationary-phase *S. aureus*, against which cornerstone anti-staphylococcal antibiotics, such as vancomycin and daptomycin, fail [30]. Considering that the clinical breakpoint for the use of nitroxoline against *E. coli*-associated uncomplicated urinary tract infections (UTIs) is 16 μg/ml [31], this activity spectrum presents several opportunities for repurposing nitroxoline against other bacterial pathogens.

Nitroxoline has traditionally been classified as bacteriostatic, even in urine [32]. Recent evidence demonstrates concentration- and species-dependent bactericidal activity, particularly against *A. baumannii* including carbapenem-resistant strains, one of the hardest-to-treat bacterial pathogens [12,29,30,33]. Future studies are needed to elucidate which bacterial determinants underlie this species specificity.

Nitroxoline shows promising activity against mycobacteria, both for multidrug-resistant *M. tuberculosis* complex (MIC_90_ 4 μg/ml) [34], and *Mycobacterium abscessus* complex isolates [35]. Earlier work had shown bacteriostatic activity at 10 μM against *Mycobacterium bovis* BCG, moderate bactericidal activity at higher concentrations, and—importantly—activity against bacilli in their hypoxic dormant state, a property relevant to the treatment of latent tuberculosis [36]. Whether urinary concentrations achieved with standard dosing are sufficient for mycobacterial clearance in urinary tuberculosis remains to be investigated.

A key aspect of nitroxoline’s spectrum that requires further investigation is its impact on the gut microbiome, which should be minimised to prevent side effects and resistance. Encouragingly, the only human study, conducted in children, reported no significant effect [37]. While nitroxoline’s positive effects on cecal flora have been more extensively studied in chickens for growth promotion [38], a deeper characterisation of these effects in humans is essential to support its clinical repurposing.

### Beyond growth inhibition and killing: antibiofilm activity

Nitroxoline exhibits broad-spectrum biofilm-eradicating activity across major bacterial pathogens, including *P. aeruginosa* [39], multidrug-resistant *A. baumannii* and *S. aureus* [30], as well as fungi like *Candida* and *Trichosporon*, particularly in combination with amphotericin B and caspofungin [40].

In *P. aeruginosa*, this activity has been linked to iron and zinc chelation from the biofilm matrix, producing reticulate rather than compact biofilm structures [39]. This extracellular chelation contrasts with the intracellular metal accumulation observed in planktonic cells, suggesting context-dependent mechanisms (Figure 2). Several mechanisms could reconcile these observations with nitroxoline’s proposed role as a copper and zinc ionophore in *E. coli* [12], *S. aureus* [15], and *M. tuberculosis* [17]. We propose four mechanisms that could account for both. First, the stability of nitroxoline-zinc or copper complexes could be pH-dependent, enabling metal release only within the cell, thereby causing metal intoxication. Second, even if these complexes remain stable, they may exert biological effects comparable to those of free metals, for example by being sensed as metal overload or directly inducing toxicity. Third, these actions may be species-specific, reflecting differences in metal uptake or export systems. Fourth, the effects may be concentration dependent, as the antibiofilm activity in *P. aeruginosa* was described for sub-MIC concentrations, whereas increased intracellular zinc in *E. coli* was observed at MIC levels.

Overall, it should be noted that the antibiofilm evidence is not uniform: while some studies report potent eradication activity [30,39], others—including work on carbapenem-resistant *A. baumannii* [29]—demonstrate reduction but not complete eradication of established biofilms. These discrepancies likely reflect differences in experimental conditions, nitroxoline concentration relative to MIC, strain background, and the distinction between inhibition of biofilm formation and eradication of mature biofilms—variables that future studies should address systematically.

## Resistance

The remarkably low clinical resistance, despite decades of use, is one of the most attractive features for nitroxoline’s repurposing. Mechanistically, resistance is primarily mediated by mutations in genes encoding efflux pumps and their regulators. In *E. coli*, low-level nitroxoline resistance is conferred by mutations in the transcriptional repressor *emrR* that upregulate the EmrAB-TolC efflux pump [12,41,42]. Higher-level resistance requires additional mutations—including *marA* mutations and *IS* element insertions upstream of *lon*, which further increase *tolC* expression [41].

A resistance mechanism conserved in *E. coli* and *Klebsiella pneumoniae* is represented by mutations in the pleiotropic regulator *envZ* [12,42]. Metabolomic and proteomic analyses revealed that nitroxoline resistance imposes major fitness costs, affecting metabolism, reducing motility, and diminishing *in vivo* virulence in a zebrafish infection model [42], which is likely to limit resistance selection in clinical settings.

Despite the clinical rarity of nitroxoline resistance, some of the mechanisms identified through *in vitro* resistance evolution were also identified in the clinics. For example, mutations in the efflux pump regulator *oqxR*, frequently detected in *in vitro-*evolved resistant *K. pneumoniae* strains, were also found in a patient undergoing a four-month prophylaxis regimen with nitroxoline [12]. Such clinical data are rare and limited to the small selection of countries where nitroxoline is used: global resistance surveillance should follow a broader clinical use of nitroxoline worldwide.

Beyond efflux pumps, resistance mechanisms seem distinct in *A. baumannii*, where oxidative stress defence mechanisms appear altered in resistant strains [12]. The functional significance of these findings requires further mechanistic studies.

## Combination therapy

Nitroxoline extensively interacts with antibiotics at the level of growth inhibition, often attributable by OM disruption: in *E. coli* it synergises with bulky antibiotics, individually not effective against Gram-negative bacteria as blocked by the OM (e.g. azithromycin, vancomycin, rifampicin) [12]. Nitroxoline also synergised with outer-membrane-targeting antibiotics such as polymyxins, resensitising colistin-resistant *Enterobacteriaceae* both *in vitro* and *in vivo* in *Galleria mellonella* [12] and mouse infection models [43]. Such an effect is evident independently of the specific colistin resistance mechanism, but was markedly stronger (up to four-fold decrease in MIC) for *E. coli* and *K. pneumoniae mcr*-*1*-positive strains [12]. A direct binding of nitroxoline to the catalytic centre of MCR-1 has been recently demonstrated in *E. coli*, where the colistin-nitroxoline combination perturbed bacterial metabolic pathways, provoked oxidative damage, and suppressed the development of colistin resistance [43] (Figure 2). This synergy was also reported independently of MCR-1 against highly colistin-resistant *P. aeruginosa*, enhancing membrane permeability, eradicating biofilms and persister cells, and completely suppressing bacterial motility [13].

While no strong interaction with quinolones or tetracyclines was observed in *E. coli* [12], nitroxoline synergised with ciprofloxacin [44] and tetracycline [45] in enteric pathogens such as *Bacillus cereus*, *Enterococcus faecalis*, *Listeria monocytogenes*, *Shigella flexneri*, and *Vibrio parahaemolyticus*. Intriguingly, the nitroxoline–ciprofloxacin combination showed antagonism towards *Bifidobacterium* species—a property that, if confirmed *in vivo*, could represent a microbiome-sparing advantage [44].

Antagonisms should be considered when proposing combinatorial regimens: in *E. coli* nitroxoline has been reported to antagonise several aminoglycosides, possibly because of its potential damage of Fe-S-cluster containing proteins responsible for the generation of the proton-motive force [12], which is necessary for aminoglycoside uptake. However, in *E. faecalis* strains nitroxoline synergised with gentamicin, inhibiting bacterial adhesion and causing morphological changes [46]. The mechanistic basis of this species-(and possibly strain-)specific interaction remains to be investigated.

The interaction between nitroxoline and beta-lactams is more complex. While extensive antagonism has been shown in the beta-lactam-sensitive *E. coli* BW25113 strain, nitroxoline and derivatives of its 8-hydroxyquinoline scaffold have been shown to inhibit metallo-beta-lactamases (MBLs), like NDM-1 and VIM-1 *in vitro* [14,47], with inhibitory activity correlating with the Fe^3+^ binding affinity of the derivative [14] (Figure 2).

Altogether, nitroxoline can resensitise Gram-negative bacteria to classic Gram-positive-targeting antibiotics and MBL-harbouring strains to beta-lactams, holding promise as adjunctive therapy to counteract both intrinsic and acquired resistance.

## Towards clinical repurposing

### Experimental *in vivo* models

A key bottleneck to nitroxoline’s repurposing is the scarcity of *in vivo* monotherapy studies, whereas such evidence is present for combinations or nitroxoline-inspired analogues. The effectiveness of the nitroxoline–colistin combination has been shown against colistin-sensitive and -resistant *K. pneumoniae* and *E. coli* strains in *Galleria mellonella* [12] and in a mouse peritonitis model [43]. Promising *in vivo* efficacy has been shown for the nitroxoline derivative ASN-1733, which outperformed the parent compound in mouse sepsis, thigh infection, and abdominal infection models [14]—a finding that both validates the antibacterial potential of the nitroxoline scaffold in systemic infection and highlights the pharmacokinetic limitations of the parent compound.

Given nitroxoline’s established safety profile *in vitro* [12,13] and *in vivo* [48] and the promising *in vitro* data reviewed above, well-designed animal studies—particularly UTI models using clinically relevant dosing—are essential to bridge the gap between *in vitro* activity and clinical translation.

### Pharmacokinetics

Knowledge of nitroxoline’s pharmacokinetic profile is limited, a consequence of its development long before the advent of standardised regulatory requirements [49]. Nitroxoline undergoes extensive hepatic conjugation, primarily to nitroxoline sulfate, and is excreted predominantly in the urine. Following administration, nitroxoline is rapidly metabolised, with only minimal levels of the parent compound detectable owing to extensive conversion into conjugated metabolites [3,32,49].

Urinary concentrations are substantially higher than plasma levels, consistent with its clinical use in UTI, but the short plasma half-life and rapid excretion severely limit tissue penetration and systemic applications [49]. Modern PK studies using approved formulations in relevant patient populations—particularly the elderly, patients with renal impairment, and those receiving combination therapy—are urgently needed.

### Clinical use: state of the art

Nitroxoline was introduced into clinical use in the 1960s, at least in Germany, where it is licensed to treat UTIs [48]. Despite more than five decades of clinical use [50], it remains used only in a handful of countries, including Germany and several Eastern European countries.

The clinical evidence supporting nitroxoline in uncomplicated UTI, though not vast by modern standards, is encouraging. An individual patient data meta-analysis encompassing four prospective, randomised clinical studies and 466 female patients demonstrated non-inferiority of nitroxoline compared with cotrimoxazole and norfloxacin, with bacteriuria eradication rates exceeding 90% in per-protocol analyses [48]. A more recent qualitative review synthesising 21 publications reached broadly similar conclusions, confirming nitroxoline as a potent broad-spectrum agent with high eradication rates in women with uncomplicated lower UTIs [3]. Consistent with these data, the 2017 German interdisciplinary S3 guideline now recommends nitroxoline as the first-line therapy for non-severe community-acquired uncomplicated UTI [51].

These historical data sets have since been validated by modern, real-world evidence. Notably, the prospective, multicentre NitroxWin study confirmed a clinical success rate of 86.3% in female patients treated for acute uncomplicated cystitis, while simultaneously demonstrating a 100% susceptibility rate to nitroxoline among all evaluated *E. coli* urinary isolates [52].

Not all patient populations seem to benefit equally from treatment with nitroxoline. In a prospective cohort study of hospitalised geriatric patients with lower UTI, a seven-day course of oral nitroxoline failed to achieve microbiological eradication. Microbiologic success at day 12 was only reached in one of nine evaluable patients. This population was old (median age 84.5 years), carried more comorbidities, and included a high proportion of complicated UTIs [53], suggesting that the bacteriostatic urinary activity of nitroxoline may be insufficient in an acute clinical context for complex patients. However, when shifted from acute treatment to long-term prophylaxis for recurrent UTIs, nitroxoline appears highly effective in complex demographics, as shown by the recent ProNitrox study. This study evaluated long-term prophylaxis (typically 3–6 months) in a cohort comprising up to 48% geriatric patients, nearly half of whom were multimorbid, and 31.6% whom had structural urinary complications. Despite these compounding risk factors, the breakthrough infection rate was low (13%) [54].

Across all studies, nitroxoline is generally well tolerated; gastrointestinal adverse events are the most common complaint, and their frequency is comparable to that observed with cotrimoxazole or norfloxacin in controlled trials [48], a profile further mirrored in recent field data where 96% of acute-use patients scored its tolerability as ‘good’ or ‘very good’ [52].

## Conclusions

In recent years, a convergence of factors has brought nitroxoline back into the spotlight: rising rates of antimicrobial resistance among uropathogens, the dwindling pipeline of new antibiotics for Gram-negative infections, and a broader movement towards drug repurposing.

Nitroxoline occupies an unusual position in the antimicrobial landscape: a drug with more than fifty years of clinical history, remarkably preserved activity, and a multimodal mechanism of action. However, several mechanistic gaps remain: nitroxoline’s interaction with metals seems dependent on the metal considered, and cannot be fully explained by a simple chelation model, depriving cells of metals, but may also include an ionophore model. It remains unclear to what extent this is dependent on metal oxidation state, concentration, extracellular testing conditions or the bacterial intracellular milieu, beyond the known dependence on pH [8]. The downstream consequences of such metal deprivation and intoxication are also complex and species-specific. Further mechanistis studies are required to understand the underlying reasons for the differential bacteriostatic or bactericidal activity of nitroxoline.

Several challenges must be addressed before nitroxoline can fulfil its potential. First, the pharmacokinetic data gap is critical: modern studies characterising absorption, tissue distribution, and dose-exposure relationships in relevant patient populations are overdue. Second, *in vivo* antibacterial efficacy data remain limited, particularly for monotherapy, and well-designed animal infection models are needed. Third, clinical trials beyond uncomplicated UTI—including complicated UTI, prostatitis, infections caused by carbapenem-resistant *A. baumannii*, and potentially urinary tuberculosis—would define the drug’s true therapeutic niche. Fourth, the combination strategies reviewed here, particularly with colistin and aminoglycosides, warrant clinical evaluation in the setting of multidrug-resistant UTIs.

Finally, the regulatory landscape is a practical barrier. Nitroxoline’s limited geographic approval reflects its pre-modern development rather than any deficiency in efficacy or safety. In an era of antimicrobial resistance, where few new antibiotics are entering clinical practice, established drugs with preserved activity deserve renewed regulatory and clinical attention. Nitroxoline, with its unique mechanism, broad spectrum, low resistance rates, and demonstrated safety, is a compelling candidate for that effort.

## Perspectives

- Rising antimicrobial resistance and the costs of marketing new antibiotics have renewed interest in repurposing older agents such as nitroxoline, which combines decades of clinical use with retained activity against multidrug-resistant pathogens.
- Nitroxoline acts on bacterial growth, resistance and biofilms through multiple mechanisms involving metal-dependent effects. Key mechanistic and pharmacokinetic questions remain unresolved to bridge the gap between *in vitro* and *in vivo* efficacy and expand therapeutic applications.
- Future work should focus on modern pharmacokinetic studies, *in vivo* efficacy models, and clinical trials in complicated and drug-resistant infections, including evaluation of combination therapies and broader regulatory approval.

## Open Access

Open access for this article was enabled by the participation of ETH Zurich in an all-inclusive Read & Publish agreement with Portland Press and the Biochemical Society.

## Abbreviations

BP: breakpoint
CRAb: carbapenem-resistant A. baumannii
ESBL: extended-spectrum β-lactamase
OM: outer membrane
LPS: lipopolysaccharide
MBEC: minimum biofilm eradication concentration
MBL: metallo-beta-lactamase
MCR: Mobilized Colistin Resistance
MDR: multidrug-resistant
MDR-TB: multidrug-resistant tuberculosis
MIC: minimal inhibitory concentration
MRSA: methicillin-resistant *Staphylococcus aureus*
NDM: New Delhi Metallo-β-lactamase
OM: outer membrane
TMP/SMX: trimethoprim/sulfamethoxazole
UTI: urinary tract infection
UPEC: uropathogenic E. coli
VIM: Verona Integron-encoded Metallo-β-lactamase
